# The CcdB toxin is an efficient selective marker for CRISPR-plasmids developed for genome editing in cyanobacteria

**DOI:** 10.17912/micropub.biology.000512

**Published:** 2022-01-12

**Authors:** Martin Menestreau, Raphaël Rachedi, Véronique Risoul, Maryline Foglino, Amel Latifi

**Affiliations:** 1 Aix Marseille Univ, CNRS, IMM, LCB, Laboratoire de Chimie Bactérienne, Marseille, France

## Abstract

Cyanobacteria, the only prokaryotes able of oxygenic photosynthesis are important primary producers that play a key role in the fields of agriculture, aquatic ecology and environmental protection. Their versatile metabolism makes them interesting candidates for various biotechnological applications. Recently, a great progress has been made in the field of their genetic manipulations by the development of CRISPR-based approaches. However, most of the available plasmids are rather difficult to manipulate, which renders their use challenging. In this study, we used the CcdB toxin as a selection marker to improve Cpf1-based plasmids designed for genome-editing in cyanobacteria. Our results demonstrate that this selection increased the rate of success of plasmid construction, and thus of genome editing.

**Figure 1. The design of a CRISPR-plasmid conditionally expressing the ccdB gene and its use for deleting the hetR gene:**
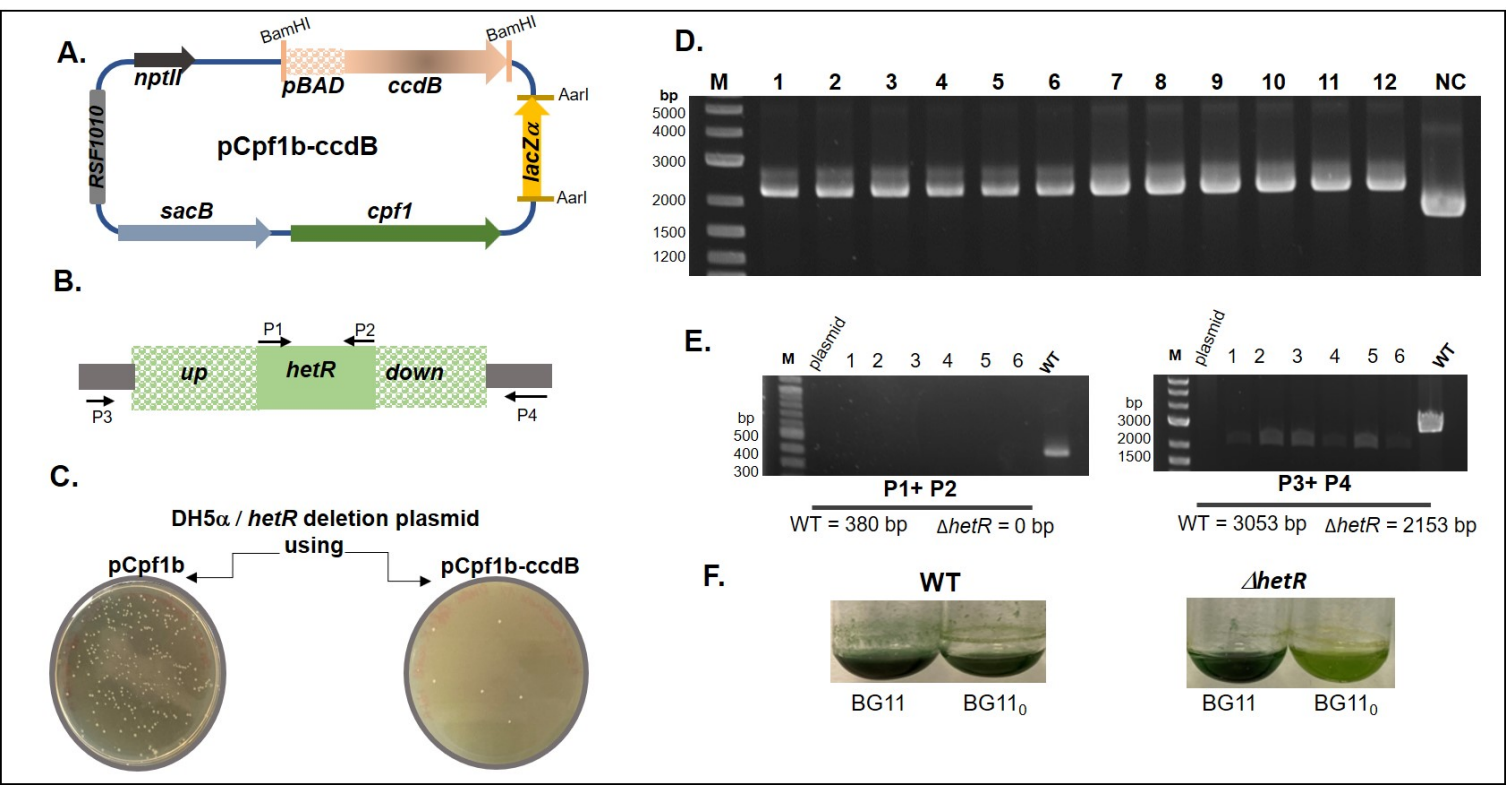
**A.** The *ccdB* -expressing plasmid developed in this study (RSF1010: replication origin, *nptII* : kanamycin/neomycin resistance marker, *pBAD* : the arabinose operon promoter, *ccdB* : the CcdB toxin coding sequence, *lacZa* : allows the identification of recombinant clones during the cloning of the spacer *cpf1* : the gene encoding the Cpf1 nuclease, *sacB* : the gene conferring sensitivity to sucrose). **B** . Schematic representation of the *hetR* gene and the upstream and downstream regions used to construct the recombination platform. The primers used for analyzing the deletion of *hetR* are indicated. The up and down boxes designate the sequences upstream and downstream to *hetR* used as template for the homologous recombination. The grey regions belong to the chromosome and are not present on the pCpf1b-ΔhetR plasmid. **C.** Comparison of the number of recombinant clones obtained after cloning either in the pCpf1b original plasmid (left) or in the plasmid harboring the *ccdB* gene (right) **. D.** PCR analysis of the cloning of *hetR* RP in the pCpf1b *–* ccdB plasmid. (M): stands for the DNA molecular size marker and the sizes of some bands are indicated. (1-12): PCR results obtained for 12 clones from four independent experiments. (NC): PCR result obtained for the pCpf1b *–* ccdB plasmid. **E.** PCR analysis of the genome of six *Nostoc* recombinant clones chosen randomly. The left image shows the PCR analysis results when P1 and P2 primers were used. The right image shows the PCR results when P3 and P4 primers were used. (M): DNA molecular size marker. (plasmid): pCpf1b-ΔhetR plasmid, which was used as a negative control. The sizes of the amplicons expected for the wild-type strain (WT) and for the *hetR* deletion mutant are indicated under each image. 1-6 stand for the 6 *Nostoc* recombinant analyzed clones. **F** . Phenotype of a delta *hetR* strain: the strains were grown either in the presence of combined nitrogen (BG11 medium) or 48 hours in its absence (BG11 _0_ medium). The WT strain was used as a positive control. The Δ *hetR* strain was yellowish when incubated 48 hours in BG11 _0_ indicating its inability to sustain growth in this medium. The *pBAD-ccdB* gene fusion was also inserted in the pCpf1-sp plasmid (see text for details). The two plasmids obtained in this study are available for the scientific community.

## Description

Recently, several CRISPR-based plasmids for editing cyanobacterial genomes have been developed. They derive mostly from vectors containing the low-copy number RSF1010 replication origin, and are of large size (> 10 kB). For instance, a CRISPR editing plasmid expressing the Cpf1 nuclease from *Francisella novicida* has been developed and shown to be efficient for editing cyanobacterial genomes (Ungerer and Pakrasi 2016). Later, this plasmid has been improved, notably by introducing the *sacB* counter-selection marker to optimize the loss of the editing plasmid once the genome-editing achieved (Niu *et al.* 2019). These plasmids are powerful tools, as their use has significantly decreased the time required to obtain mutants in cyanobacterial model strains. However, it has been largely reported that cloning in plasmids bearing the RSF1010 replication origin is difficult using techniques relying both on restriction enzymes, and approaches based on isothermal assembly. The copy number of RSF1010-type plasmids has been evaluated to be between 0.5 to 11.8 in *Escherichia coli* (Jahn *et al.* 2016), which explains the low yield of DNA obtained after plasmid extraction, and ineffective use in cloning procedures. In addition, the ability of RSF1010 plasmids to self-mobilize has also been suggested to explain their poor use as cloning-vectors (Taton *et al.* 2014). For all these reasons, we decided to first test our ability to manipulate the Cpf1-CRISPR plasmids by deleting the *hetR* gene of *Nostoc* PCC 7120 because the phenotype of the mutant is well defined (see below). In our hands, the cloning of the spacer was not a limiting-step due to the possibility of identification of positive clones by alpha-complementation (Ungerer and Pakrasi 2016). However, the insertion of the recombination platform (RP) **–** which contains the regions upstream and downstream to the gene to be deleted- was rather difficult, as a high number of negative clones are usually obtained (our success rate was less than 5%). In order to improve the cloning-success with this plasmid, we sought to introduce a screen that would allow direct selection of recombinant clones that have integrated the RP. To do this, we modified the plasmids pCpf1b (conferring resistance to kanamycin/neomycin) and pCpf1 *–* sp (conferring resistance to spectinomycin) (Niu *et al.* 2019) by introducing the *ccdB* gene encoding a toxin that inhibits the DNA gyrase activity (Bernard and Couturier 1992). For this, the *ccdB* gene under the control of the *pBAD* promoter was amplified from the NM580 strain (Battesti *et al.* 2015) and cloned into the BamHI site of pCpf1b and pCpf1-sp plasmids. The XL1 Blue strain producing the anti-toxin CcdA was used for this experiment to inhibit the action of CcdB (Bernard and Couturier 1992). Over 100 colonies screened only 2 had the *ccdB* gene. A map of the obtained recombinant plasmid when pCpf1b was modified, is shown in Figure 1A. For the use of this plasmid for gene editing, the RP must be cloned between the BamHI sites, thus replacing the *ccdB* gene. This cloning must be performed in a strain that does not express the *ccdA* anti-toxin gene and arabinose must be added to the medium to induce expression of the toxin-encoding gene. Therefore, only clones that have integrated the RP are viable as the negative clones are counter-selected by the CcdB-induced lethality.

To analyze the efficiency of *ccdB* , we used the resulting pCpf1b *–* ccdB plasmid to delete the *hetR* gene of *Nostoc* PCC 7120. This cyanobacterium is able to fix dinitrogen thanks to the nitrogenase enzyme (Elhai and Wolk 1990). In this bacterium, nitrogen fixation relies on the ability to differentiate micro-oxic cells, that host the oxygen-sensitive nitrogenase (Flores and Herrero 2010). HetR is the master regulator of cell differentiation and is therefore essential when dinitrogen is the only nitrogen source available (Buikema and Haselkorn 2001). The scheme presented in Figure 1B describes the upstream and downstream regions used as the RP sequence to construct the *hetR* deletion mutant. The RP for *hetR* deletion was inserted in the pCpf1b-ccdB plasmid digested by BamHI (Figure 1A). For the selection of the *E. coli* recombinant clones, arabinose was added to the selective medium. The images of Figure 1C compare the total number of clones obtained when the cloning was achieved using the original (pCpf1b) or the modified (pCpf1b-ccdB) plasmids in a representative experiment. The results of Figure 1D show the analysis of recombinant clones chosen randomly during four independent experiments. The presence of the RP sequence was analyzed by PCR using the RP fw and rv primers and the pCpf1b *–* ccdB plasmid was used as a negative control. The expected amplicon size is 1734 pb for the pCpf1b *–* ccdB plasmid and 2186 pb in the case of successful RP cloning. As observed in Figure 1D, all the analyzed clones were positive which indicates that the *ccdB* gene is indeed an efficient selective marker. One of these plasmids was used to insert the *hetR* specific spacer. To optimize the targeting of the gene to be edited and its cleavage by Cpf1, we used the recently reported ChopChop software (Labun *et al.* 2019) to choose the spacer sequence (see Methods). The spacer sequence was cloned as explained in the Methods section. Positive colonies were identified by the alpha-complementation procedure. One of the resulting pCpf1-ccdB *-Δ* hetR plasmids was introduced by conjugation in *Nostoc* to delete the *hetR* gene. The PCR analysis of 6 clones obtained 15 days after the conjugation step demonstrated that all of them displayed the deletion (Figure 1E). As expected for a *hetR* mutant, the obtained strains were unable to sustain growth in the absence of combined nitrogen (Figure 1F). Additionally, we have used these improved plasmids to delete numerous other genes in a variety of on-going projects and a similar rate of success for the cloning of the RP sequences was obtained. We therefore conclude that, compared to their parental plasmids, the pCpf1-ccdB plasmids significantly optimize genome editing procedure in cyanobacteria.

## Methods

**Methods**

**Cyanobacterial strains growth**

*Nostoc* PCC 7120 and derivatives were grown in BG11 medium (Rippka R 1979) at 30 °C under continuous illumination (40 µE m ^-2^ s ^-1^ ). When appropriate, media were supplemented with neomycin at a final concentration of (50 μg mL ^−1^ ). Conjugation of *Nostoc* was performed as described in reference (Cai and Wolk 1990). To assess the phenotype of the *hetR* deletion mutant the strains were in BG11 _0_ (BG11 without sodium nitrate).

**Construction of the *ccdB* -editing plasmid**

The *pBAD-ccdB* sequence was amplified from the genome of the NM580 strain using the *ccdB fw* and *ccdB rv* primers. The In-Fusion technique (Takara) was used to insert the obtained amplicons in the pCpf1b and pCpf1-sp which were first linearized by BamH1. The XL1-Blue strain was used for transformation and the recombinant clones were analyzed by PCR, using the *ccdB fw* and the *ccdB rv* primers. Positive clones were further analyzed by sequencing.

**Construction of the pCpf1-ccdB *-DhetR* plasmid**

1kpb upstream and downstream of the *hetR* gene were amplified using the *hetR up fw* / *hetR up rv* and *hetR down fw* / *hetR down rv* primers, respectively. The two resulting amplicons were inserted in the BamHI site of the pCpf1-ccdB plasmid using the In-Fusion approach. Recombinants clones were obtained in the DH5α strain grown in the presence of arabinose at a final concentration of 0,1%. The recombinant plasmid obtained was analyzed by sequencing and named pCpf1-ccdB/RP

The spacer sequence was designed using the ChopChop software (Labun *et al.* 2019). The size of the primers was set to 22 nucleotides and the 5’-PAM sequence was designed TTN, as advised by Niu T et. al 2019 (Niu *et al.* 2019). The spacer- *hetR* fw and spacer- *hetR* rv primers were annealed at 95°C to obtain the *hetR* spacer, which was then inserted in the AarI site of the pCpf1b-ccdB/RP. The plasmids were introduced in the DH5alpha strain and recombinant clones were identified on selective medium containing Xgal at a final concentration of 80 μg/ml and isopropyl β-D-1-thiogalactopyranoside (IPTG) at a final concentration of 0,5mM. Positive clones were further analyzed by sequencing.

## Reagents

***Escherichia coli* strains**

**Table:** 

**Strain name**	**Genotype**	**Source**
NM580	MG1655 *lacI* -T1T2- *zeo ^R^* -pBR *placO* -kan-pBAD- *ccdB* . Mini-l-Red:: *tet ^R^*	MA Lab
XL1-Blue	*recA1 endA1 gyrA96 thi-1 hsdR17 supE44 relA1 lac* [F´ *proAB lacIqZ∆M15* Tn10 (Tetr)]	Stratagene
DH5α	F ^–^ *endA1* *glnV44* *thi-1* *recA1* *relA1* *gyrA96* *deoR* *nupG* *purB20* φ80d *lacZ* ΔM15 Δ( *lacZYA-argF* )U169 *, hsdR17* ( *r _K_* ^–^ *m _K_* ^+^ ), λ ^–^	Invitrogen

**Plasmids**

**Table:** 

**Name**	**Description**	**Source**
pCfp1b	Cpf1-based CRISPR plasmid with kanamycin resistance marker and SacB counter selection marker	Addgene **(** #122187)
pCfp1-sp	Cpf1-based CRISPR plasmid with spectynomycin resistance marker and SacB counter selection marker	Addgene **(** #122186)

**Sequences of the primers used in this study**

**Table:** 

**Primer name**	**Sequence (from 5’ to 3’)**	**Experiment**
*ccdB fw*	TAGATCTCATGGATCCAATTGTCTGATTCGTTACCAATT	Cloning of the *pBAD-ccdB* sequence in the pCpf1b and pCpf1-sp plasmids
*ccdB rv*	TTGCCATTGCGGATCCTTATATTCCCCAGAACATCAGGTT	
spacer *hetR* fw	agatAGCATAAGTTACCCAGCAATCT	Cloning of the *hetR* spacer
spacer *hetR* rv	agacAGATTGCTGGGTAACTTATGCT	
*hetR up fw*	ATATCTAGATCTCATGGATCCTGGTATTGGCAAAATACAAAATCCC	Cloning of the *hetR* upstream region: obtaining the RP
*hetR up rv*	CTCTGGGTGCATTACAAATAGTTGAATAGCACGC	
*hetR down fw*	TATTTGTAATGCACCCAGAGTGAATAAAAGTACT	Cloning of the *hetR* upstream region: obtaining the RP
*hetR down rv*	CGTTGTTGCCATTGCGGATCCTCGCGGATGATGGTATTAAGCT	
RP fw	CCTTTTGTATTAGTAGCCGG	Analysis of the insertion of the RP sequence
RP rv	TCTAGAGTCGAGCGCAA	
P1	ATCTGATCAAGCGTCTTGG	Analysis of the deletion of *hetR*
P2	TTCTTCACTTGTGAGGCTTG	
P3	AAACATTGCCCAAATATTCG	
P4	TTCTTCACTTGTGAGGCTTG	
